# *Mycobacterium tuberculosis* infection and tuberculosis disease in the first decade of life: a South African birth cohort study

**DOI:** 10.1016/S2352-4642(24)00256-6

**Published:** 2024-12

**Authors:** Fernanda Bruzadelli Paulino da Costa, Mark P Nicol, Maresa Botha, Lesley Workman, Ricardo Alexandre Arcêncio, Heather J Zar, Leonardo Martinez

**Affiliations:** a University of São Paulo, São Paulo, Brazil; b Department of Paediatrics and Child Health, Red Cross War Memorial Children's Hospital, South African Medical Research Council Unit on Child and Adolescent Health, University of Cape Town, Cape Town, South Africa; c School of Biomedical Sciences, Marshall Centre, University of Western Australia, Perth, WA, Australia; d Department of Epidemiology, School of Public Health, Boston University, Boston, MA, USA

## Abstract

**Background:**

Paediatric tuberculosis leads to more than 200 000 deaths annually. We aimed to investigate the incidence of *Mycobacterium tuberculosis* infection and tuberculosis disease in the first decade of life in the Drakenstein Child Health Study (DCHS), a South African cohort in a community with high tuberculosis and HIV incidence.

**Methods:**

In this prospective birth cohort study, we enrolled pregnant women aged 18 years or older who were between 20 and 28 weeks’ of gestation in a peri-urban setting outside of Cape Town, South Africa. We followed up their children for tuberculosis until age 10 years. To measure *M tuberculosis* infection tuberculin skin tests were administered to children at age 6 months, 12 months, and then annually in children with a negative test, and at the time of a lower respiratory tract infection. Tuberculin skin test conversion was defined by an induration reaction of 10 mm or more. To measure tuberculosis disease, active surveillance was done throughout follow-up. Each episode of presumed tuberculosis disease was investigated using sputum induction, tested with Xpert MTB/RIF and liquid culture for *M tuberculosis*. Survival analyses were performed and multivariable Cox regression was used to measure factors associated with *M tuberculosis* infection or disease.

**Findings:**

Between March 5, 2012, and March 31, 2015, 1137 women and their 1143 children (248 [21·7%] of 1143 children were HIV-exposed, two [0·2%] children with HIV) were included in the analysis. Children were followed up for 8870 person-years (median follow-up 9·1 years [IQR 8·2–10·2]). The annual risk of tuberculin conversion during follow-up was 6·6 infections per 100 person-years (95% CI 5·8–7·3) but ranged from 4–9 infections per 100 person-years over the follow-up period. 98 children developed tuberculosis (1105 cases per 100 000 person-years; 95% CI 906–1347). The cumulative hazard of tuberculin conversion was 36% (95% CI 32–41) at age 8 years and the cumulative hazard of tuberculosis disease was 10% (8–12) at age 10 years. Preventive treatment was associated with a reduction in tuberculosis disease among children who had tuberculin conversion (adjusted hazard ratio 0·23 [95% CI 0·12–0·47]). Most cases of tuberculosis disease (78 [79%; 95% CI 69–86] of 98 children) occurred among children who had tuberculin skin test conversion but were not administered preventive treatment.

**Interpretation:**

In this prospective South African birth cohort, *M tuberculosis* transmission was consistently high throughout the first decade of life leading to approximately 10% of children developing tuberculosis disease. A multipronged approach to decrease paediatric tuberculosis is needed that combines preventive treatment for children at risk, reducing community *M tuberculosis* transmission, and active case finding.

**Funding:**

Bill & Melinda Gates Foundation, Medical Research Council South Africa, and National Research Foundation South Africa.

## Introduction

Tuberculosis is a leading cause of child mortality worldwide with most deaths occurring before age 5 years.^1^ Approximately 1·2 million children, half younger than 5 years, develop the disease every year.^2^, ^3^ In South Africa, 15–20% of all tuberculosis cases are likely to occur in children; generally, the proportion of all tuberculosis cases that occur in children is higher in settings with large tuberculosis epidemics.^2^, ^4^, ^5^, ^6^ Despite the high tuberculosis mortality among children and recognised increased disease risk, longitudinal community cohorts that follow children for tuberculosis disease over time are infrequent.^7^, ^8^

Delineating the burden of *Mycobacterium tuberculosis* infection and disease among children is difficult, especially in resource-constrained settings with a high burden of tuberculosis.^8^ Generally, surveillance in children is poorly resourced and in young children, most tuberculosis disease is paucibacillary adding further complexity to diagnosis.^8^, ^9^ In settings with high HIV prevalence and undernutrition, diagnosis might be even more challenging since disease could present with non-specific symptoms and signs, and diagnostic performance of tests might be minimally sensitive.^10^, ^11^ Most estimates of tuberculosis disease incidence are based on data from health-care facilities and do not reflect true community-based incidence.^12^ Few studies have systematically and prospectively investigated the community burden of tuberculosis in children, or prenatal and early-life risk factors for the development of *M tuberculosis* infection and disease. Large, population-based, prospective birth cohort studies from high-burden settings with serial follow-up for *M tuberculosis* infection and disease are not widely reported but are key to evaluate the evolving epidemiological situation as children age in sub-Saharan Africa and other high-burden settings.

**Research in context**

**Evidence before this study**

We searched PubMed using two different search strings for articles published in English, from March 1, 2014, to March 31, 2024, for other birth cohorts investigating tuberculosis-related outcomes in high tuberculosis incidence settings. The search terms used were “(child* OR pediatric OR paediatric OR infant) AND (tuberculosis OR TB) AND (incidence)” for the first search and “(‘birth cohort’) AND (tuberculosis OR TB)” for the second search. Both searches were restricted to the title and abstract fields. We previously reported high rates of tuberculin skin test conversion and tuberculosis disease in young children up to age 5 years enrolled in the Drakenstein Child Health Study. We found no other community-based birth cohort studies of children evaluated for *Mycobacterium tuberculosis* infection or tuberculosis disease in the first decade of life in high tuberculosis incidence settings. Accurate longitudinal data from these settings are scarce for the incidence of *M tuberculosis* infection and primary progressive tuberculosis disease in infants and young children, and for prenatal and early-life risk factors for tuberculosis-related outcomes.

**Added value of this study**

To our knowledge, this is the first birth cohort study to prospectively investigate *M tuberculosis* infection and tuberculosis disease in the first decade of life in a setting with high tuberculosis incidence. Our results suggest a high annual risk of *M tuberculosis* infection that was largely consistent throughout childhood, ranging from 4–9 cases per 100 person-years, suggesting substantial ongoing and uninterrupted *M tuberculosis* transmission. By 8 years of age, the cumulative hazard of tuberculin skin test conversion was 36%; by 10 years of age, the cumulative hazard of tuberculosis disease was 10%. We found that the tuberculosis disease incidence was 1105 cases per 100 000 person-years over the 10-year follow-up time period; incidence was highest in the first year of life, decreasing through childhood, despite very few children with HIV and no children identified with severe malnutrition. Preventive treatment was highly effective in children who had tuberculin skin test conversion. Despite this, most eligible children were not administered preventive treatment and most tuberculosis disease occurred among children with tuberculin skin test conversion but who were not administered preventive treatment.

**Implications of all the available evidence**

Around 10% of children in this study done in a setting with a high incidence of tuberculosis developed tuberculosis disease in the first decade of life. This high incidence of tuberculosis disease was likely to be driven by the consistently high *M tuberculosis* transmission rates throughout this period. A multipronged approach to decrease paediatric tuberculosis in high-burden settings such as South Africa is needed that combines preventive treatment through effective preventive programmes for children who are at risk, reducing community *M tuberculosis* transmission, and active case finding.

We previously reported high rates of tuberculin skin test conversion and tuberculosis disease in the first 5 years of life among children enrolled in the Drakenstein Child Health Study, a South African birth cohort study.^6^ In this study, we extend this work with the aim of investigating *M tuberculosis* infection (through tuberculin skin test conversion) and tuberculosis disease in children in the first decade of life in this prospective birth cohort from a South African community with a high burden of tuberculosis. We assessed the risk and cumulative burden of *M tuberculosis* infection and disease over time and factors associated with these two outcomes.

## Methods

### Study design and participants

In this prospective birth cohort study (the Drakenstein Child Health Study), we enrolled pregnant women in a peri-urban setting outside of Cape Town, South Africa, as previously described.^6^, ^13^, ^14^, ^15^, ^16^, ^17^ Eligible women were between 20 and 28 weeks’ of gestation. Exclusion criteria for pregnant women were being younger than 18 years and intending to leave the study area within 1 year. All mothers accessed care in the public sector with a strong primary health-care programme, including a vertical HIV transmission prevention programme and well developed immunisation programme. Infants were given Bacillus Calmette–Guérin (BCG) vaccination at birth as per national policy. Women were followed through pregnancy and childbirth; thereafter, mother–child pairs were followed until children were 10 years of age.

Ethical approval was obtained from the University of Cape Town Faculty of Health Sciences Human Research Ethics Committee (reference numbers 401/2009 and 651/2013) and the Western Cape Provincial Health Research Committee. Mothers provided written informed consent at enrolment, which was renewed annually; assent was provided by child participants from 7 years of age and onwards depending on the cognitive ability of each child. We involved people with related lived experience in the study design and implementation; involvement of study participants assessing their experience of the study and their feedback was done and is ongoing.

### Procedures

Comprehensive questionnaires about maternal health were completed at enrolment during pregnancy and longitudinally. Antenatal care occurred at primary health care clinics; all births took place at the single public sector hospital in the setting. Mothers were tested for HIV during pregnancy with a HIV-1/2 rapid antibody test (Abbott Laboratories, Chicago, IL, USA). If positive, a confirmatory ELISA was done. All mothers who tested HIV-positive received antiretroviral therapy as per national guidelines and infants of those mothers were tested for HIV using PCR (Cobas AmpliPrep system; Roche Molecular Systems, Branchburg, NJ, USA) at age 6 weeks and 6 weeks after the end of breastfeeding. Birth information included birthweight and method of delivery. Follow-up visits, including clinical examinations, were done at 6, 10, and 14 weeks; 6 and 12 months; and thereafter 6 monthly. Data for environmental exposures, household characteristics, respiratory risk factors, and child symptoms were obtained every 6 months at scheduled visits. Mothers were counselled about respiratory symptoms at every visit and advised to attend the study site or contact study staff between scheduled study visits whenever the child developed a cough or difficulty breathing. Active surveillance for lower respiratory tract infections and tuberculosis was done throughout follow-up, as described previously.^18^, ^19^

To measure *M tuberculosis* infection, tuberculin skin tests (TSTs) were done at the 6-month visit and 12-month visit, and then annually, and at the time of a lower respiratory tract infection in children with a previous negative TST, as previously described.^6^, ^13^, ^14^, ^15^, ^16^, ^17^ TSTs were considered positive (ie, TST conversion), if induration was at least 10 mm following recommendations by South Africa's Department of Health and global and regional guidelines.^20^, ^21^ Children who tested TST positive, if not diagnosed with tuberculosis disease, were referred to local clinics for tuberculosis preventive treatment. Trained study staff collected induced sputum specimens in duplicate for liquid mycobacterial culture or PCR (Xpert MTB/RIF; Cepheid, Sunnyvale, CA, USA) from all children with a positive TST, and from children who were presumed to have or who had been clinically diagnosed with tuberculosis. A chest radiograph was taken in all children investigated for tuberculosis. Tuberculosis disease was diagnosed and defined based on standardised international guidelines and included confirmed (microbiologically positive) and unconfirmed (clinically diagnosed) categories.^22^

A lower respiratory tract infection was diagnosed according to WHO criteria, including any child presenting with a general danger sign or with cough or difficulty breathing, and age specific tachypnoea or lower chest wall retractions.^15^ Smoking during pregnancy was assessed using the Fagerström questionnaire. Maternal smoking was documented through interviews conducted with mothers during follow-up visits. Exposure to tuberculosis cases was assessed at each follow-up visit. We enquired about the presence of tuberculosis disease in the home or among close family or community members, and a child was considered to have had contact with tuberculosis if such contact was reported at any of these visits.

### Statistical analysis

All mother–child pairs were included in the analysis. Mothers were excluded if there was a pregnancy loss or women were lost to follow-up during the antenatal period. We summarised continuous variables as medians with IQR and categorical variables using proportions.

There were two primary outcomes. The first primary outcome was *M tuberculosis* infection as represented by TST conversion in the follow-up period. For this outcome, time-to-event began at birth and ended on the date of a positive TST result. A child was assumed to have a negative TST at birth. For TST conversion, follow-up was censored after 8 years due to the low number of observations. Conversion rates were calculated as the number of conversions per 100 person-years during the follow-up time period. The second primary outcome was incident tuberculosis disease during follow-up. We used a composite outcome including both confirmed and unconfirmed tuberculosis. For tuberculosis, time-to-event began at birth and ended when a child was diagnosed with disease. Follow-up was censored at death, loss to follow-up, or Feb 1, 2024. Tuberculosis disease incidence was calculated as the number of diagnoses per 100 000 person-years during the follow-up time period.

Risk of *M tuberculosis* infection or tuberculosis disease throughout the cohort follow-up was calculated using cumulative hazard curves. We compared children with TST conversion with those without TST conversion and analysed subsequent incidence of disease through to the end of study. Smoothed hazard plots showing instantaneous risk of the outcome were generated across the first decade of life (ie, risk that the outcome will occur at a given age conditional on the outcome not having occurred before that). Kaplan-Meier curves were used to estimate tuberculosis-free survival in the first decade. We specified a kernel function to be used in calculating the weighted Epanechnikov kernel-density smoothing of the estimated hazard over age and time.^23^ This function is calculated from a fully non-parametric model and, therefore, a minimum number of events are needed before a credible hazard function estimate can be computed. This process was done for both TST conversion and tuberculosis disease for the overall sample; we repeated this process for children with TST conversion but who were not administered preventive treatment.

For each outcome, we used Cox proportional hazard models for multivariable modelling and results are presented as hazard ratios (HRs). For each outcome of interest (TST conversion and tuberculosis disease), we present univariable models (indirect effects) and multivariable models (direct effects, after adjustment for the effects of confounding variables). In separate regression models for each outcome, we analysed factors associated with TST conversion (representing *M tuberculosis* infection) and incident tuberculosis disease. For each outcome, we present univariable and multivariable models. Variables that showed a statistical association with the outcome were included in each multivariable model. We evaluated correlations among variables using polychoric correlation coefficients, which measure correlation between ordered levels where the latent trait can be considered continuous and normally distributed. HRs and adjusted HRs (aHRs) with corresponding 95% CIs are presented. We used 95% CIs to assess statistical significance in all models. The likelihood ratio test was used to decide on final Cox regression models.

### Role of the funding source

The funders of the study had no role in study design, data collection, data analysis, data interpretation, or writing of the report.

## Results

**Figure 1:**
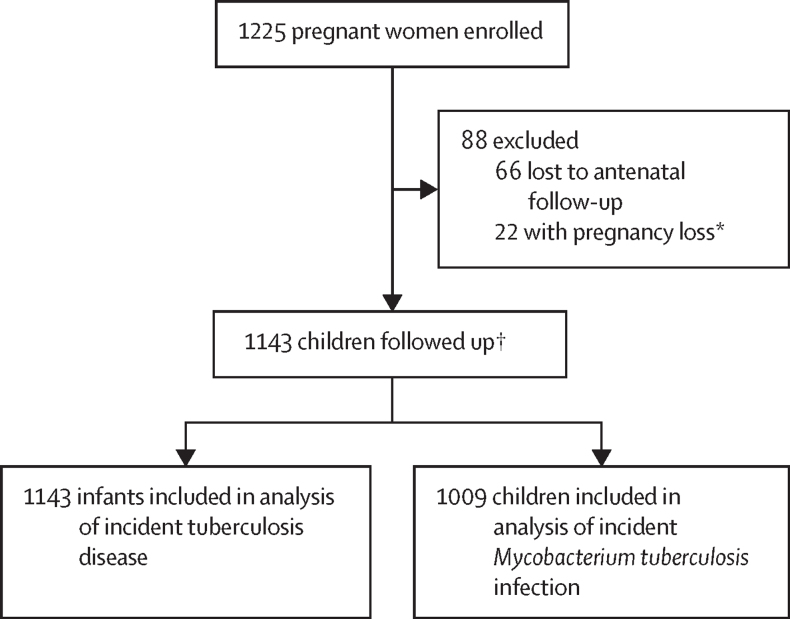
Study flow *Loss of pregnancy due to miscarriage, stillbirth, or intrauterine death (23 infants [including one set of twins]). †Including four pairs of twins and one set of triplets.

Between March 5, 2012, and March 31, 2015, 1225 pregnant women were enrolled; 88 (7·2%) women were excluded due antenatal loss to follow-up or pregnancy loss, thus 1137 (92·8%) women gave birth to 1143 children who were included in the analysis (figure 1). Among 1143 children, cumulative follow-up was 8870 years (median 9·1 years [IQR 8·3–10·2]). Retention in the cohort was high; at 1 year 89·9% (n=1028), at 5 years 85·9% (n=982), and at 10 years 85·7% (n=980) of the cohort continued to be followed up (appendix pp 2, 3).

Pregnant women were a median age of 26 years (IQR 22–31) at antenatal enrolment. 323 (28·3%) of 1142 women self-reported smoking during pregnancy. 248 (21·7%) of 1143 mothers were living with HIV and all were receiving antiretroviral therapy. Only two (0·2%) of 1143 children had HIV (table 1). Low birthweight was identified in 176 (15·4%) of 1143 children. During follow-up, almost half of children (523 [45·8%] of 1143) developed a lower respiratory tract infection. Documented tuberculosis exposure during the study period was also common, occurring in 505 (44·2%) of 1143 children.

**Table 1:** Sociodemographic and clinical characteristics of participants included in the Drakenstein Child Health Study

		**Participants (n=1143)**	**Children with no tuberculosis-related outcome (n=845)**	**Tuberculin skin test conversion without disease (n=200)**	**Tuberculosis disease (n=98)**
**Child characteristics**					
Sex*					
	Male	586 (51·3%)	426 (50·4%)	105 (52·5%)	55 (56·1%)
	Female	557 (48·7%)	419 (49·6%)	95 (47·5%)	43 (43·9%)
Low birthweight (<2·5 kg)		176 (15·4%)	721 (85·3%)	168 (84·0%)	20 (20·4%)
Preterm birth		192 (16·8%)	138 (16·3%)	31 (15·5%)	23 (23·5%)
Breastfed at any time		842/1067 (78·9%)	593/771 (76·9%)	166/199 (83·4%)	83/97 (85·6%)
HIV exposure		248 (21·7%)	197 (23·3%)	35 (17·5%)	16 (16·3%)
HIV infection		2 (0·2%)	2 (0·2%)	2 (0·2%)	0
Any LRTI		523 (45·8%)	353 (41·8%)	107 (53·5%)	63 (64·3%)
LRTI with hospital admission		189 (16·5%)	120 (14·2%)	41 (20·5%)	28 (28·6%)
Close contact with tuberculosis		505 (44·2%)	335 (39·6%)	109 (54·5%)	61 (62·2%)
Isoniazid preventive treatment		93 (8·1%)	28 (3·3%)	55 (27·5%)	10 (10·2%)
Ancestry†					
	Black African	632/1142 (55·3%)	485/844 (57·5%)	112 (56·0%)	35 (35·7%)
	Mixed ancestry	510/1142 (44·7%)	359/844 (42·5%)	88 (44·0%)	63 (64·3%)
**Maternal characteristics**					
Age at time of childbirth, years		26 (22–31)	26 (22–31)	25 (22–30)	26 (22–30)
Education					
	No education	84/1141 (7·4%)	56/843 (6·6%)	16 (8·0%)	12 (12·2%)
	Primary school only	609/1141 (53·4%)	444/843 (52·7%)	107 (53·5%)	58 (59·2%)
	Some secondary school	375/1141 (32·8%)	291/843 (34·5%)	58 (29·0%)	26 (26·5%)
	Finished secondary school	73/1141 (6·4%)	52/843 (6·2%)	19 (9·5%)	2 (2·1%)
Self-reported smoking during pregnancy		323/1142 (28·3%)	220/844 (26·1%)	98 (34·2%)	42 (42·9%)
Self-reported maternal smoking (any timepoint)		400 (35·0 %)	400 (35·0%)	78 (39·0 %)	50 (51·0%)
**Household characteristics**					
Crowding (persons per house)					
	≤2	166 (14·5%)	138 (16·3%)	18 (9·0%)	10 (10·2%)
	3–4	428 (37·5%)	319 (37·8%)	77 (38·5%)	32 (32·7%)
	≥5	550 (48·0%)	388 (45·9%)	105 (52·5%)	56 (57·1%)
Household income (ZAR per month)					
	<1000 (approximately $US50)	386/1142 (33·8%)	267/844 (31·6%)	81 (40·5%)	38 (38·8%)
	1000–5000 (approximately $50–250)	596/1142 (52·2%)	440/844 (52·1%)	103 (51·5%)	53 (54·1%)
	>5000 (approximately $250)	160/1142 (14·0%)	137/844 (16·2%)	16 (8·0%)	7 (7·1%)
Any smoker in the household		864 (75·6%)	601 (71·1%)	175 (87·5%)	88 (89·8%)

Data are n (%), n/N (%), or median (IQR). LRTI=lower respiratory tract infection. ZAR=South African Rand.

* Information on sex was collected by the study team at the time of childbirth.

† Participants self-reported ancestry.

**Figure 2:**
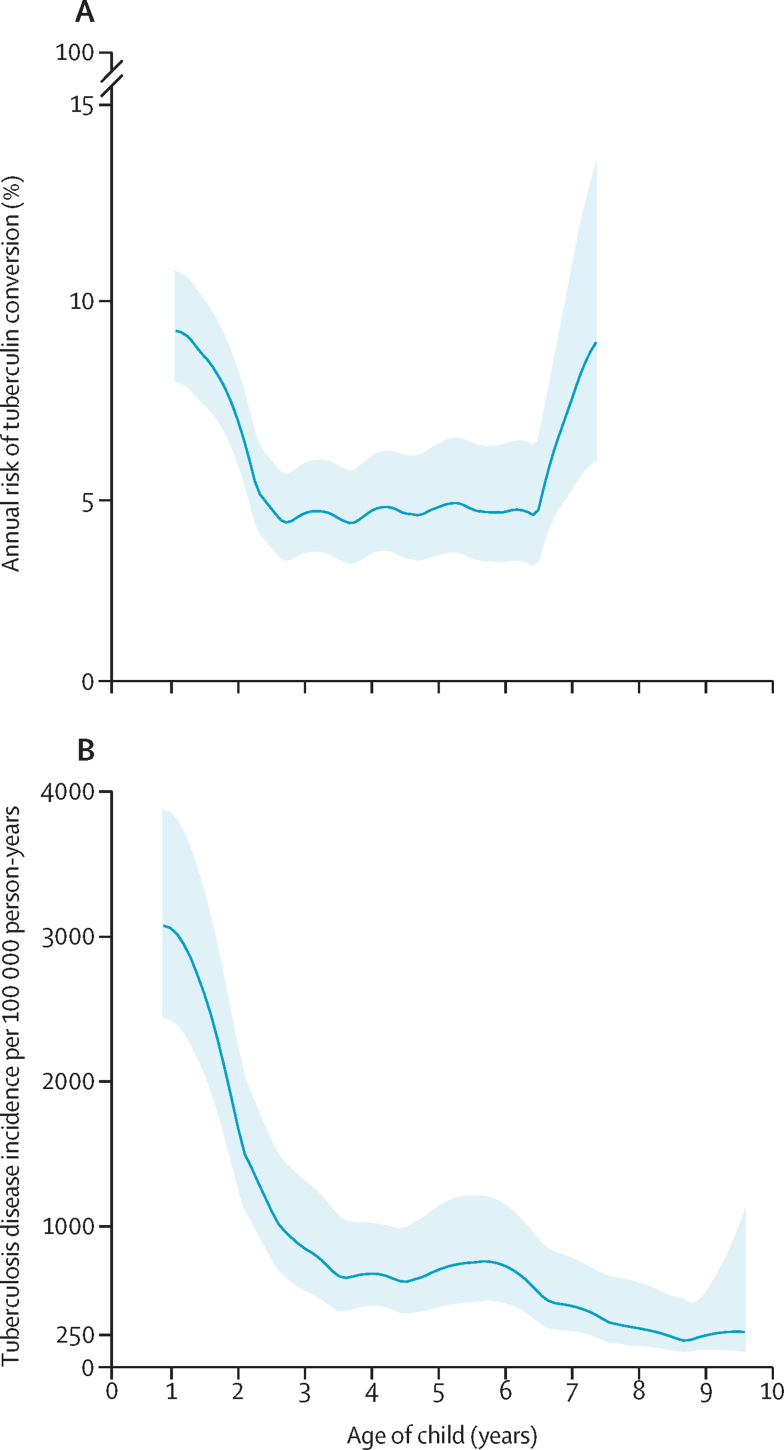
The annual percentage risk of tuberculin skin test conversion (A) and tuberculosis disease incidence per 100 000 person-years (B) Panel A describes the probability that a tuberculin skin test conversion will occur at a given age conditional on no tuberculin skin test conversion having occurred before that. Because this was a non-parametric model, we were unable to compute results preceding the first observed event. Moreover, some minimum number of events are needed before an instantaneous hazard function can be computed. Many children did not have tuberculin skin test results after the 8-year visit; therefore, panel A stops before the end of total study follow-up. Panel B shows tuberculosis disease incidence per 100 thousand person-years over the entire cohort through a hazard function or the probability that a tuberculosis diagnosis will occur at a given age conditional on no tuberculosis diagnosis having occurred before that. This specifies a kernel function to be used in calculating the weighted kernel-density estimate smoothing of the estimated hazard over age. The solid line in each graph represents modelled estimates and the shaded bands are 95% CIs.

Overall, 4302 TSTs were performed in 1009 (88·3%) of all 1143 children followed up in the study between 2012 and 2023. 287 (6·7%) of 4302 TSTs were positive, indicating TST conversion, and these positive results were distributed over the follow-up (appendix p 3). Across the follow-up period, the annual risk of TST conversion overall was 6·6 (95% CI 5·8–7·3) per 100 person-years. The risk of tuberculin conversion ranged from 4·4% (95% CI 3·5–5·7) to 9·3% (8·0–10·8; figure 2A), with the greatest annual risk occurring at 1 year (9·3% [8·0–10·8]) and 8 years (8·9% [6·0–13·4]), although the sample size was small at age 8 years (figure 2A). The 8-year cumulative hazard of TST conversion was 36% (95% CI 32–41). Risk factors associated with TST conversion included being male (aHR 1·2 [95% CI 1·0–1·6]), household cigarette smoke exposure (1·7 [1·2–2·5]), households with more than five people (1·3 [1·1–1·7]), known exposure to an individual with tuberculosis (1·2 [1·0–1·6]), and developing a lower respiratory tract infection (1·2 [1·0–1·6]; table 2).

**Table 2:** Risk factors for tuberculin skin test conversion during the first decade of life in the Drakenstein Child Health Study

		**Tuberculin skin test conversion (n=287)**	**Incidence per 100 person-years**	**HR (95% CI)**	**aHR (95% CI)**
Sex					
	Female	132	5·6	1 (ref)	1 (ref)
	Male	155	7·5	1·3 (1·0–1·6)	1·2 (1·0–1·6)
Low birthweight (<2·5 kg)					
	No	239	6·3	1 (ref)	..
	Yes	48	7·7	1·2 (0·9–1·7)	..
Any smoker in the house					
	No	256	3·5	1 (ref)	1 (ref)
	Yes	31	7·2	2·1 (1·4–3·1)	1·7 (1·2–2·5)
Crowding (people per household)					
	≤5	168	5·6	1 (ref)	1 (ref)
	>5	119	8·3	1·5 (1·1–1·9)	1·3 (1·1–1·7)
Household exposure to tuberculosis case					
	No	125	5·5	1 (ref)	1 (ref)
	Yes	162	7·6	1·4 (1·1–1·7)	1·2 (1·0–1·6)
Any LRTI*					
	No	128	5·6	1 (ref)	1 (ref)
	Yes	159	7·5	1·3 (1·1–1·7)	1·2 (1·0–1·6)

Variables that showed a statistical association were included in the final model. For tuberculin conversion shown in this table, sex, any smoker in the house, crowding, household exposure to a tuberculosis case, and any LRTI were included in the multivariable model. HR=hazard ratio. aHR=adjusted hazard ratio. LRTI=lower respiratory tract infection.

* A LRTI was diagnosed according to WHO criteria, including any child presenting with a general danger sign or with cough or difficulty breathing, and age specific tachypnoea or lower chest wall retractions.

98 (8·6%) of 1143 children developed tuberculosis disease, most of whom (81 [83%] of 98) were under 5 years (appendix p 4). All cases, with the exception of one case of tuberculous meningitis, presented as pulmonary tuberculosis. There was a slight increase in cases between June, 2020, and April, 2021 (appendix p 8) during the COVID-19 pandemic after the extreme lockdown in South Africa. 90 (92%) of 98 children presumed to have tuberculosis had at least one induced sputum for culture and Xpert, among whom 12 (12%) of 98 were microbiologically confirmed, all of which were rifampicin-sensitive tuberculosis cases. 86 (88%) of 98 children had unconfirmed tuberculosis disease, of whom 66 (77%) cases were classified by a combination of criteria, TST conversion, and history of close contact; 12 (14%) cases were TST positive only, seven (8%) were diagnosed based on clinical criteria (cough, weight loss), and one (1%) case had a history of close household contact (appendix p 5). All children who had tuberculosis clinically diagnosed had improvement in clinical symptoms or signs with treatment on follow-up.

**Figure 3:**
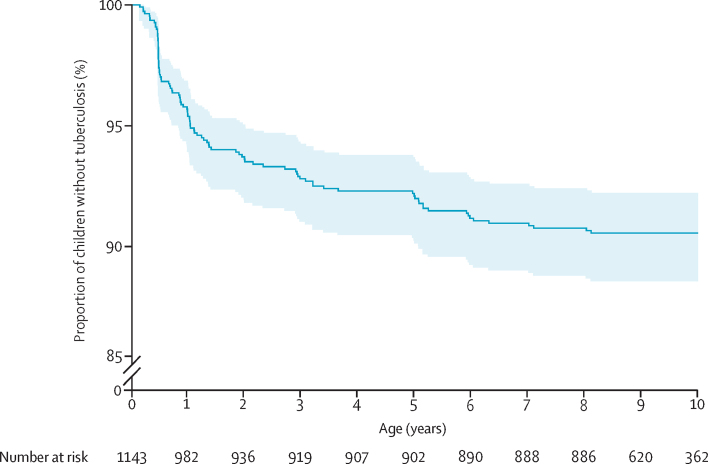
Tuberculosis-free survival in the first decade of life Kaplan–Meier estimates of tuberculosis-free survival in children from the Drakenstein Child Health Study. The solid line represents modelled estimates and the shaded bands are 95% CIs. The hazard ratio and CI were calculated using a Cox proportional-hazards model. There was no indication of non-proportional hazards.

Across the entire follow-up period, the risk of tuberculosis disease was 1105 cases per 100 000 person-years (95% CI 906–1347). The incidence of tuberculosis among infants aged 1 year was 3053 cases per 100 000 person-years (95% CI 2441–3860), decreasing to 1618 cases per 100 000 person-years (1240–2181) at 2 years and 284 cases per 100 000 person-years (176–607) at 8 years (figure 2B). The 1, 5, and 10-year cumulative hazard of tuberculosis was 4% (95% CI 3–6), 8% (7–10), and 10% (8–12), respectively (figure 3). Previous positive TST was a risk factor for tuberculosis disease (aHR 27·6 [95% CI 13·3–57·4]; table 3).

**Table 3:** Risk factors for tuberculosis disease during the first decade of life in the Drakenstein Child Health Study

		Tuberculosis events (n=83)*	**Incidence per 100 000 person-years**	**HR (95% CI)**	**aHR (95% CI)**
Sex					
	Female	40	906	1 (ref)	..
	Male	43	966	1·1 (0·7–1·7)	..
Low birthweight (<2·5 kg)					
	No	65	858	1 (ref)	..
	Yes	18	1398	1·6 (0·9–2·8)	..
Any smoker in the house					
	No	10	635	1 (ref)	..
	Yes	73	1001	1·6 (0·8–3·4)	..
Crowding (people per household)					
	≤5	48	805	1 (ref)	..
	More than 5	35	1207	1·5 (0·9–2·4)	..
Household exposure to tuberculosis case					
	No	34	738	1 (ref)	1 (ref)
	Yes	49	1151	1·6 (1·0–2·5)	1·3 (0·8–1·9)
Any LRTI†					
	No	35	755	1 (ref)	1 (ref)
	Yes	48	1136	1·5 (1·0–2·4)	1·2 (0·8–1·9)
Tuberculin skin test conversion					
	No	8	118	1 (ref)	1 (ref)
	Yes	75	3556	30·0 (14·5–72·1)	27·6 (13·3–57·4)

HR=hazard ratio. aHR=adjusted hazard ratio. LRTI=lower respiratory tract infection.

* Any tuberculosis diagnosis made clinically or microbiologically, excluded cases who had a tuberculosis diagnosis before the first episode of LRTI, or up to 30 days after.

† A LRTI was diagnosed according to WHO criteria, including any child presenting with a general danger sign or with cough or difficulty breathing, and age specific tachypnoea or lower chest wall retractions. In this table, the variables household exposure to tuberculosis case, any LRTI and tuberculin skin test conversion were included in the multivariable model.

Among the 287 children with TST conversion during follow-up, 63 (22%) converted after age 5 years. Preventive treatment was administered for 64 children, of whom nine (14%) developed tuberculosis disease (appendix p 6). Of the 223 children who did not receive preventive treatment, 78 (35%) developed tuberculosis disease. In a multivariable model including only children with tuberculin conversion, children who received preventive treatment had a lower risk of tuberculosis disease than children who did not receive preventive treatment (aHR 0·23 [95% CI 0·12–0·47]; appendix p 7). 78 (79%; 95% CI 69–86) of 98 tuberculosis cases occurred among children who had TST conversion but were not administered preventive treatment.

## Discussion

To the best of our knowledge, this is the first birth cohort study to prospectively investigate *M tuberculosis* infection and tuberculosis disease in the first decade of life in an area with a high tuberculosis burden. Our results suggest a high annual risk of *M tuberculosis* infection, which was largely consistent throughout childhood, ranging from 4–9%. We found that the tuberculosis disease incidence was 1105 cases per 100 000 person-years throughout the first decade of life; incidence was highest in infants, dropping through childhood, despite a low number of children with HIV. More than 10% of all children included in this community-based cohort developed tuberculosis disease during the follow-up period representing a high disease burden that is not only associated with acute illness but also long-term morbidity.^15^

Although paediatric tuberculosis has recently received greater attention globally,^7^ few studies have investigated the risk of *M tuberculosis* infection and disease in the first decade of life. The high disease burden in this South African setting is concerning. These results are likely to reflect availability through the study of active case finding and screening with multiple annual study visits and additional visits provided by the study team during episodes of illness and the large burden of tuberculosis in socioeconomically disadvantaged communities. Approximately 90% of children with presumed tuberculosis had one or more induced sputum collections suggesting the study team had sufficient concern for tuberculosis and sufficient testing infrastructure. Although this active surveillance is likely to have led to high case detection, it is possible that cases of tuberculosis might be unreported.^2^, ^12^ If 10% of children in high-burden settings are acquiring tuberculosis in the first decade of life, this represents a population at high risk for poor outcomes in young adulthood and later life. These include impacts on familial income and post-tuberculosis lung health as reported in this cohort followed up to 5 years of age and in other studies.^15^, ^24^, ^25^, ^26^, ^27^ Follow-up of this cohort into adolescence to understand long-term health trajectories will be an important next step.

We found that the risk of TST conversion remained broadly similar throughout follow-up, ranging from 4–9% depending on the age of the child. These results are largely consistent with other early childhood cohorts using QuantiFERON conversion as an endpoint.^28^ In this study, a slightly increased annual risk of *M tuberculosis* infection was identified in the first few years of life (from 6–8%), followed by a reduction in risk (approximately 4–5%), followed again by an increase in risk between age 6 and 8 years. The rise in *M tuberculosis* infection risk later in childhood might be due to increased exposures as children begin school. However, lower statistical power might also have led to a drop in precision. Generally, our study findings show higher annual risk of *M tuberculosis* infection compared with studies using testing from one timepoint.^29^, ^30^, ^31^ These studies have consistently estimated annual rates of infection of less than 5%, even in settings with high tuberculosis rates. Our findings also support the argument that the force of *M tuberculosis* infection is likely to be underestimated in high-burden settings.^31^ We found the cumulative hazard of TST conversion to be 36% at age 8 years, which suggests these children are infected several times throughout the first decade of life due to the intensity of community transmission. Both infection and progression to disease are expected to increase in early and late adolescence:^32^ if reinfection occurs, how it impacts tuberculosis progression will be paramount to understanding disease patterns later in life.

Despite the consistently high annual risk of *M tuberculosis* infection, the rate of progression to tuberculosis disease decreased with age in our cohort, decreasing to around 200 cases per 100 000 person-years by 10 years. This has been observed in other historical cohorts using *M tuberculosis* infection tests at a single timepoint. For example, follow-up of a large cohort in Puerto Rico of children who were TST positive, found that the risk of tuberculosis disease peaked in the first 5 years of life, then decreased later in childhood, followed by a later increase in adolescence.^33^ Explanations for the observed patterns in age-specific disease risk from our results, in combination with past findings, might be multifactorial. Protection from disease with age (even as the annual risk of infection remains consistent) might suggest immune maturation over time, protection as children are reinfected with *M tuberculosis*, or early-life immunological hits, such as following viral infection, which facilitate disease.^13^, ^34^, ^35^

We identified several risk factors for both TST conversion and disease. These included a recent close exposure to a tuberculosis case. There is a substantial risk of *M tuberculosis* infection and disease among contacts exposed to patients with tuberculosis in a variety of settings and the incidence of incident tuberculosis is highest in the first years after exposure.^32^ Crowding is also well documented as an important risk factor, as it increases the probability of *M tuberculosis* exposure among participants and can also reflect low socioeconomic conditions. An increase in tuberculosis was observed during the first year of the COVID-19 pandemic, after lifting of stringent lockdown regulations in South Africa. The pandemic had a detrimental effect on global control of childhood tuberculosis for several reasons,^36^, ^37^ but in the case of this cohort, children continued to be followed up, and studies to identify COVID-19 also took place, which might have increased the likelihood of identifying tuberculosis.

Use of isoniazid preventive treatment substantially reduced risk of progression from infection to disease (aHR 0·23). This might be an underestimate of true protection of preventive treatment for disease progression but is similar to estimates from case-contact cohorts (aHR 0·09–0·20).^38^ Factors that might have led to an underestimate of effectiveness include adherence of preventive treatment, which could not be assessed in this investigation. Other reasons could include *M tuberculosis* reinfection that might occur after the end of preventive treatment. Importantly, only 10% of children who developed tuberculosis disease received preventive treatment. This highlights an important challenge in tuberculosis programmes where provision of preventive treatment is of low priority.

Our study had limitations. First, we used a standard 10 mm induration cutoff to indicate a positive TST, which might overestimate or underestimate *M tuberculosis* infections. We used this cutoff to be conservative, however boosting of repeated skin tests or impact from BCG vaccination might lead to false positivity. Children who had a positive TST were no longer tested annually which is likely to minimise boosting. The use of QuantiFERON testing might have limited bias from boosting (although the data on this is mixed);^39^, ^40^ however, the need for serial blood samples over several years, laboratory testing, and cost are not feasible in this setting. Second, the priority of TST within our population was to identify conversion, not reversions. Due to this, we did not retest participants after they tested positive. People who have TST reversion (ie, a positive test becomes negative) are likely to be a heterogenous group, however a proportion of reversions might represent *M tuberculosis* clearance; in this case, our estimates of the annual risk of *M tuberculosis* infection might be overestimated.^41^ Third, due to a variety of reasons, including refusal of testing and large numbers of participants testing TST positive during follow-up (and who were therefore were censored in subsequent analyses), few participants remained in the analysis of TST conversion between age 8 and 10 years. Therefore, our ability to calculate patterns in *M tuberculosis* infection during these later follow-up years is limited. Fourth, our risk factor analyses are not likely to be generalisable to communities in settings with a low prevalence of tuberculosis.

In conclusion, we report epidemiological findings from, to our knowledge, the first prospective birth cohort to measure the burden of *M tuberculosis* infection and disease in the first decade of life from a hyperendemic setting with a high burden of both HIV and tuberculosis. We identified a high tuberculosis disease incidence that dropped with age. Despite this, we found consistently high annual rates of *M tuberculosis* infection, suggesting considerable ongoing and uninterrupted transmission. A multipronged approach to decrease paediatric tuberculosis in high-burden settings such as South Africa is needed that combines preventive treatment for children at risk, reducing community *M tuberculosis* transmission, and active case finding.

### Contributors

### Data sharing

The data used for this analysis can be made available upon reasonable request once all relevant substudies from the Drakenstein Child Health Study are reported and completed. The data dictionary can be made available on request from the corresponding author.

## Declaration of interests

We declare no competing interests.
