# Supplementary material for: Mycobacterium tuberculosis infection and tuberculosis disease in the first decade of life: a South African birth cohort study
Source: Lancet Child Adolesc Health. 2024 Dec;8(12):891–9. doi: 10.1016/S2352-4642(24)00256-6 (PMC11579303; doi:10.1016/S2352-4642(24)00256-6)

THE LANCET
Child & Adolescent Health

Supplementary appendix

This appendix formed part of the original submission and has been peer reviewed. We post it as supplied by the authors.

Supplement to: da Costa FBP, Nicol MP, Botha M, et al. *Mycobacterium tuberculosis* infection and tuberculosis disease in the first decade of life: a South African birth cohort study. *Lancet Child Adolesc Health* 2024; published online Nov 5. https://doi.org/10.1016/S2352-4642(24)00256-6.

Table of Contents

Table S1. Cascade of care for screening, tuberculin skin testing, and preventive treatment, Drakenstein Child Health Study

Table S2. Participant retention and tuberculin skin testing, Drakenstein Child Health Study

Table S3. Description of persons diagnosed with tuberculosis, Drakenstein Child Health Study

Table S4. Basis for tuberculosis disease diagnosis among unconfirmed cases, Drakenstein Child Health Study

Table S5. Characteristics of tuberculin converters who were and were not administered preventive treatment, Drakenstein Child Health Study

Table S6. Statistical models assessing the relationship between preventive treatment and tuberculosis progression among children who tuberculin converted, Drakenstein Child Health Study.

Figure S1. Timepoints of positive tuberculin skin tests and tuberculosis diagnoses, Drakenstein Child Health Study

Table S1. Cascade of care for screening, tuberculin skin testing, and preventive treatment, Drakenstein Child Health Study

	**n**	**%**
Children intended for screening	1,143	100.0
Children tested	1,009	88.1
Tests were read	989	86.3
At least one result	988	86.2
Tested positive	287	25.1
Isoniazid preventive treatment	93	8.1

Table S2. Participant retention and tuberculin skin testing, Drakenstein Child Health Study

Time point, (age in months)	Children retained	Tuberculin skin test carried out	Tuberculin conversion
0	1,143	0	0
1-5	1,069	305	24
6-11	1,028	1,048	101
12-23	1,003	472	29
24-35	994	462	33
36-47	987	432	17
48-59	982	348	20
60-71	980	338	18
72-83	980	218	11
84-95	980	271	11
96-107	980	251	14
108-119	980	5	0
>=120	980	67	9
**Total**		**4,217***	**287**

*85 TST done without date

Table S3. Description of persons diagnosed with tuberculosis, Drakenstein Child Health Study

**Characteristics**	**n**	**%**
Episodes	107	
Children with tuberculosis	98	
Case definition		
Confirmed tuberculosis	12	12.2
Unconfirmed tuberculosis	86	87.8
Basis for starting tuberculosis medication		
Strong contact history (adult in the same household)	27	27.6
Other contact history	11	11.2
Chest X-ray suggestive of tuberculosis	32	32.7
Chronic cough (> 2 weeks)	26	26.5
Weight loss / failure to gain weight	37	37.8
Tuberculin skin test positive	77	78.6
Other diagnostic tests	7	7.1
Treatment		
Standard tuberculosis treatment	87	88.8
Tuberculosis meningitis treatment	1	1.0
Other treatment	10	10.2
First induced sputum 1 done	90	91.8

Table S4. Basis for tuberculosis disease diagnosis among unconfirmed cases, Drakenstein Child Health Study

**Characteristics**	**n**	**%**
Unconfirmed tuberculosis	86	87.8
Basis for tuberculosis diagnosis		
Tuberculin skin test positive	12	14.0
Clinical symptoms	7	8.1
Strong contact history (adult in the same household)	1	1.2
Chest X-ray suggestive of tuberculosis	0	0.0
Combination of several criteria	66	76.7

Table S5. Characteristics of tuberculin converters who were and were not administered preventive treatment, Drakenstein Child Health Study

Characteristics of tuberculin converters (N=287)	Preventive treatment	
	Yes (N=64)	No (N=223)
	n (%)	n (%)
Age of tuberculin conversion: median months (range)	10 (5-88)	24 (4-123)
Sex		
Male	38 (59.4)	117 (52.4)
Female	26 (40.6)	106 (47.5)
Study site		
Mbekweni	28 (43.8)	113 (50.7)
TC Newman	36 (56.2)	110 (49.3)
HIV exposure	13 (20.3)	36 (16.1)
Maternal education		
No education	7 (10.9)	21 (9.4)
Primary school only	37 (57.8)	119 (53.4)
Some secondary school	18 (28.1)	64 (28.7)
Finished secondary school	2 (3.2)	19 (8.5)
Household income (ZAR per month)		
<1,000 (approx. $50)	25 (39.1)	92 (41.2)
1,000 - 5000 (approx. $50- $250)	34 (53.1)	113 (50.7)
>5,000 (approx. $250)	5 (7.8)	18 (8.1)
Ethnicity		
Black African	29 (45.3)	113 (50.7)
Mixed ancestry	35 (54.7)	110 (49.3)

Table S6. Statistical models assessing the relationship between preventive treatment and tuberculosis progression among children who tuberculin converted, Drakenstein Child Health Study.

Statistical Model	Adjustment	Hazard Ratio	95% Confidence Intervals
Model 1 (base model)	None	0.35	0.18-0.70
Model 2	Study site	0.31	0.16-0.62
Model 3	Study site, sex	0.30	0.15-0.60
Model 4	Study site, sex, age at conversion	0.24	0.12-0.48
Model 5	Study site, sex, age at conversion, maternal HIV	0.23	0.12-0.47

Figure S1. Timepoints of positive tuberculin skin tests and tuberculosis diagnoses, Drakenstein Child Health Study

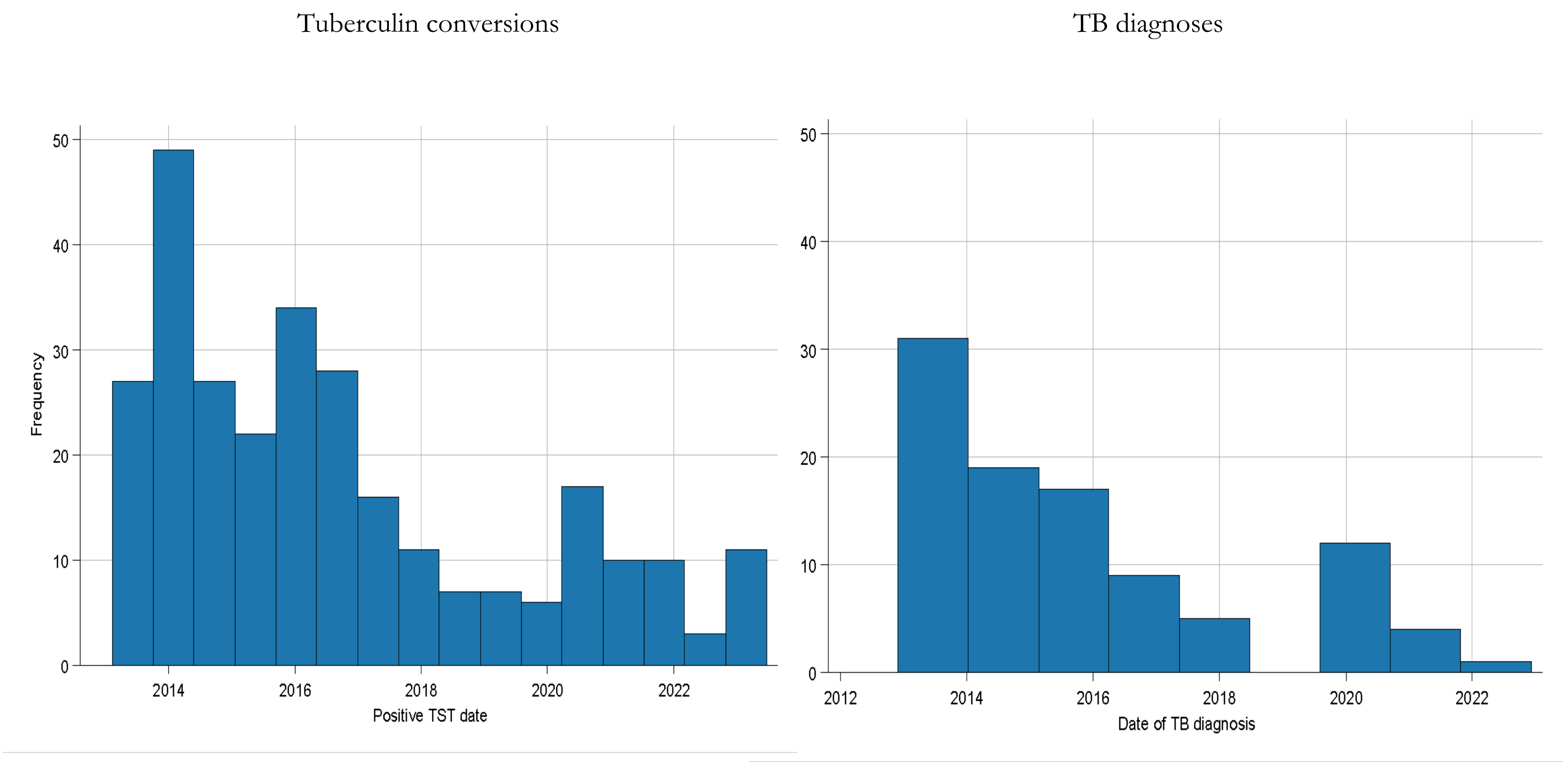

Supplement: Supplementary appendix [file mmc1.pdf]
